# Omphalocele, Exstrophy of Cloaca, Imperforate Anus, and Spinal Defects Complex: A Case Report

**DOI:** 10.31729/jnma.8048

**Published:** 2023-04-30

**Authors:** Suraj Singh, Anuj Kayastha, Anupama Thapa, Bijay Thapa, Sulochana Dahal

**Affiliations:** 1 Department of Pediatrics Surgery, Kanti Children's Hospital, Maharajgunj, Kathmandu, Nepal

**Keywords:** *anorectal malformations*, *bladder exstrophy*, *case reports*, *neural tube defects*, *umbilicus*

## Abstract

Omphalocele, exstrophy of cloaca, imperforate anus, and spinal defects complex is a rare malformation complex that includes omphalocele, cloaca! exstrophy, imperforate anus and spinal defects with the incidence of 1 in 200,000 to 400,000 pregnancies and is even rarer in twin gestation. The etiology of this complex is still unclear. Most cases are sporadic. Prenatal screening must be done for diagnosis and appropriate multidisciplinary management of cases. In severe cases, termination of pregnancy is considered. We present a 4-day first twin child with underdeveloped ambiguous genitalia delivered via emergency lower section cesarean section at 32^+3^ weeks of gestation with giant liver containing omphalocele, cloacal exstrophy, imperforate anus and meningocele with severe pulmonary artery hypertension and non-visualization of right kidney and ureter, absence of uterus, fallopian tubes and right ovary. Separation and repair of the cecum and bladder were done. The ladd procedure was performed. Ileostomy was created and single-stage repair of the abdominal wall was done.

## INTRODUCTION

The term OEIS (Omphalocele, Exstrophy of Cloaca, Imperforate Anus, Spinal Defects) was coined by Carey (1978) for a rare constellation of malformations.^1^ Clinical findings are numerous. The main findings are failure of fusion of the genital tubercles and pubic rami, incomplete development of the lumbosacral vertebrae with spinal dysraphism, imperforate anus, cryptorchidism and epispadias in males and anomalies of the Mullerian duct derivatives in females, and a wide range of urinary tract anomalies. Most patients have a single umbilical artery.^2^ It results from improper closure of the anterior abdominal wall and defective development of the cloaca and urogenital septum due to a defect in blastogenesis during the 4^th^ week of gestation.^1,3^

## CASE REPORT

A 4-day-old child with underdeveloped ambiguous genitalia was referred to the emergency department of the Kanti Children's Hospital with a history of anterior abdominal wall defect with protrusion of intestinal part and urinary bladder, absence of anal opening and spinal defect. The patient is the first twin delivered at 32+3 weeks of gestation by emergency lower segment cesarean section at the hospital with a birth weight of 1500 gm. Apgar score was satisfactory at 1 and 5 minutes of birth. The second twin was a female with a birth weight of 2000 gm and she was doing well. There is no history of consanguineous marriage.

**Figure 1:**
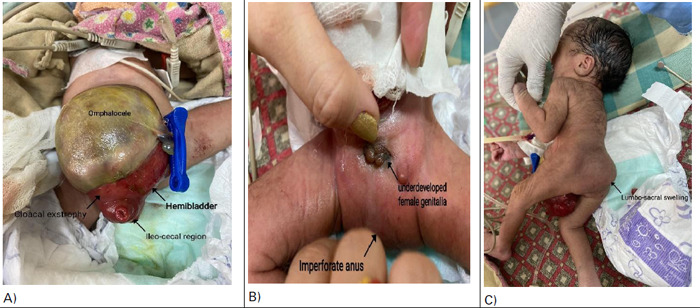
Preoperative appearance of newborn revealing A) Omphalocele, cloacal exstrophy, hemibladder and prolapsed ileocecal region, B) Under-developed female genitalia and imperforate anus and, C) lumbosacral swelling.

On examination, the general condition was fair and without pallor, icterus, cyanosis, dehydration, or edema. Respiratory rate was above the cutoff value for the age and oxygen saturation was 90-94% under the oxygen hood. Chest examination revealed subcostal retraction with bilateral conducted sound. On cardiovascular examination, first and second heart sounds were heard and also systolic murmur was heard. Abdomen and pelvic examination revealed anterior abdominal wall defect over the peri-umbilical region with midline herniation of the abdominal viscera covered by a membranous sac, and umbilical cord attached to its base and foreshortened gut fused with bladders in the lower part of the abdomen. The prolapsed cecal surface was seen while the anal opening was absent. There was a wide pubic symphysis diastasis and underdeveloped genitalia. Also, lumbosacral swelling was noticed (Figure 1).

**Figure 2:**
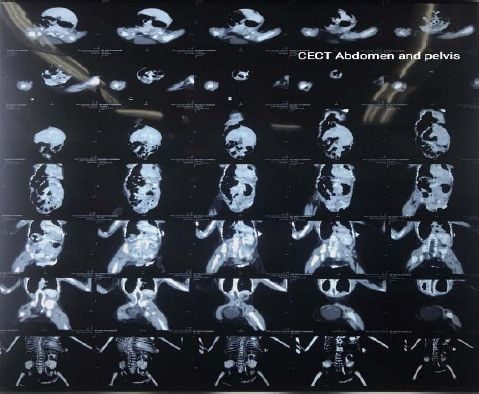
CECT film of the abdomen and revealing the congenital anomalies.

After initial resuscitation, the patient was evaluated for other associated anomalies. Echocardiography revealed tubular ductus arteriosus 2.7 mm (only systolic laminar flow). Also, atrial septal defect secundum type 4.1 mm, both left to right shunt with moderate tricuspid regurgitation with severe pulmonary artery hypertension (estimated PASP 56+10= 66 mm Hg) and mildly dilated right atrium, the right ventricle was detected. Contrast-enhanced computed tomography scan revealed a spinal defect as lumbosacral meningocele and non-visualization of the right kidney and left kidney occupying the left lower hemithorax. The patient was consulted for evaluation by a neurosurgeon, and there was a recommendation for observation without immediate neurosurgical intervention (Figure 2).

**Figure 3:**
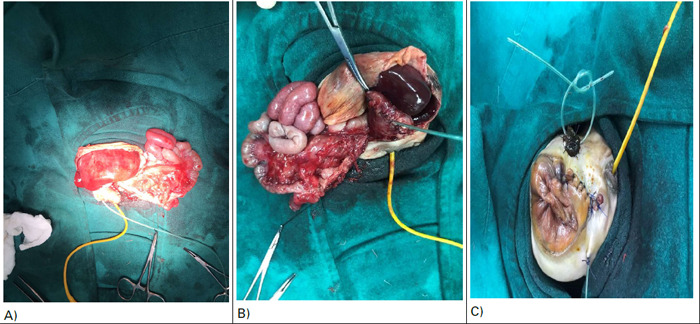
Intra-operative findings revealed A) mal-rotation of the gut, B) repair of the cecal and bladder plate and C) closure of the abdominal wall with an ileostomy.

Surgery was planned and high risks consent was taken from the parent. A midline incision over the membranous sac was made and which revealed the liver-containing protrusion of the abdominal viscera. Mal-rotation of the gut was identified. Cecum and urinary bladder were fused. The gastrointestinal tract distal to the cecum could not be appreciated. The right kidney and right ureter, uterus, fallopian tube, and right ovary could not be appreciated. Left ureteric insertion into the urinary bladder and left ovary was identified. Foley's catheter of 5 Fr was inserted into the urinary bladder. The cecal plate was separated from the bladder and the bladder tabularized with the insertion of a 4 Fr-sized feeding tube in the left ureter. The ladd procedure was done for mal-rotation of the gut. Ileostomy was created from the distal ileum in the left lower quadrant of the abdomen and the cecum was repaired. The abdomen was closed with an approximation of a membranous sac as the omphalocele was giant (Figure 3).

The patient was transferred to SICU and was kept ventilated for 2 days and went into sudden cardiopulmonary arrest.

## DISCUSSION

OEIS is the acronym for malformations complex association including omphalocele, exstrophy of bladder or cloaca, imperforate anus, and spinal defects with an incidence of 1 in 200,000 to 400,000 pregnancies. Besides these cardinal malformations, the OEIS complex has been reported to have other associated anomalies of the central nervous system, cardiovascular system, vertebrae, upper urinary tract, malrotation, defects in the vertebral column, absent appendix, lower limb abnormalities like talipes equino-valgus, single umbilical artery.^1^

In our case, the OEIS complex was associated with cardiac anomalies which consisted of tubular ductus arteriosus, atrial septal defect secundum, both left to right shunt with moderate tricuspid regurgitation with severe pulmonary artery hypertension and mildly dilated right atrium, right ventricle. Renal anomalies included non-visualization of the right kidney and ureter. Likewise, the gastrointestinal tract distal to the cecum could not be appreciated. Also, the uterus, fallopian tube, and right ovary could not be appreciated. There was wide pubic symphysis diastasis and underdeveloped ambiguous genitalia.

Although OEIS is sporadic in nature with no obvious etiology, in some cases associated chromosomal aberrations have been reported. It has been suggested that the association of OEIS with homeobox genes such as homeobox HB9 and retinoic acid and its receptors.^1^ In one of the study reported OEIS in sib from separate pregnancies concluding autosomal recessive inheritance, multifactorial, gonadal mosaicism for a dominant mutation, environmental factors.^3^ Association of OEIS with deletion of chromosome 1p36 has been reported.^4^ OEIS has also been reported in association with maternal exposure to diazepam, cigarette smoking, maternal obesity, and uterine fibroids.^3,4^

Cloacal exstrophy develops early in the first trimester secondary to a defect in the early mesoderm. Failure of migration of the lateral mesodermal folds leads to unfused inferior abdominal muscles with omphalocele and lack of fusion of the pubic bones. Failure of cloacal septation leads to the persistence of a cloaca, a rudimentary mid-gut, and an imperforate anus.^1,5^

Antenatal ultrasound helps in diagnosing and preventing fetal death with appropriate management. Major criteria for the prenatal ultrasound diagnosis of cloacal exstrophy include non-visualization of the fetal bladder, infra-umbilical abdominal wall defect, omphalocele, myelomeningocele, and minor criteria include lower extremities malformations, renal anomalies, ascites, widened pubic arches, narrow thorax, hydrocephalus, single umbilical artery. An additional sonographic feature in the prenatal diagnosis of cloacal exstrophy, calling it 'the elephant trunk-like image', represents a prolapsed terminal ileum.^5^ In our case, prenatal ultrasound was inconclusive.

Surgical repair is always challenging due to the complexity of these malformations and requires that multidisciplinary teams include neonatologists, pediatric surgeons, pediatric urologists, pediatric orthopedists, pediatric neurosurgeons, and genetic and pediatric endocrinologists.^6,7^

The goals of OEIS management are; separating the bowel from the bladders to create an intestinal stoma, closing the omphalocele, an adaptation of the bladder halves, and adequate cosmetic and functional urogenital reconstructions with preserving renal function. The surgical treatment of OEIS can be achieved in single or multiple stages with an increasing preference towards the staged approach. The single-stage management of cloacal exstrophy as a part of the OEIS complex has the potential to fail and cause problems with the outcome. The major etiology of failure is severe symphysis diastasis causing inadequate approximation of the pubic bones and tight closure of large abdominal defects leading to organ ischemia.^6^

In our case, we have separated and repaired the fused cecum and bladder with the insertion of a 4 Fr size feeding tube in the left ureter. The ladd procedure was done. Also, an ileostomy was created in the left lower abdomen. The abdomen was a closed approximation of a membranous sac.

Kaya reported a case of 1900 gm delivered by caesarian section after a 35-week twin gestation with OEIS complex had sustained staged surgical intervention and was discharged at 29 days.^6^ Neel reported a 10 years old who had undergone multiple constructive surgeries for the OEIS complex.^7^ Hence staged surgery should be considered.

Though there is various case reported around the world the exact etiology behind the malformation is still unknown, so much study needs to be done and we document the first such case from the country.
